# Identifying the long-term survival beneficiary of chemotherapy for stage N1c sigmoid colon cancer

**DOI:** 10.1038/s41598-022-21331-z

**Published:** 2022-10-07

**Authors:** Shan Liu, Yaobin Lin, Sihan Huang, Shufang Xue, Ruoyao Huang, Lu Chen, Chengyi Wang

**Affiliations:** 1 Department of Hematology-Oncology, Fujian Children’s Hospital, Fuzhou, China; College of Clinical Medicine for Obstetrics and Gynecology and Pediatrics, Fujian Medical University, Fuzhou, China; 2 Clinical Oncology School of Fujian Medical University, Fujian Cancer Hospital, Fuzhou, Fujian China; 3 Department of Hematology-Oncology, Fujian Maternity and Child Health Hospital, Affiliated Hospital of Fujian Medical University, Fuzhou, China

**Keywords:** Cancer, Gastroenterology, Oncology, Risk factors

## Abstract

Sigmoid colon cancer often has an unsatisfactory prognosis. This study explored the effect of tumor deposits (TDs) on survival, and whether their presence/absence influence individualized treatment. Data of postoperative patients with sigmoid colon cancer were extracted from the Surveillance, Epidemiology, and End Results database. Overall survival (OS) was calculated using the Kaplan–Meier method and prognostic factors were identified using Cox regression analysis and random forest (RF). The nomogram's discrimination performance was evaluated using a concordance index (C-index), integrated discrimination improvement (IDI), calibration curves, and decision-curve analysis. The N1c group showed a worse prognosis than the N0 group. For N1c patients, a combination of surgery and chemotherapy prolonged survival, compared to surgery alone; however, the chemotherapy-surgery combination did not affect the OS of patients younger than 70 years, in stage T1–2, and/or of black race. Multivariable analysis and RF presented Age, T stage, and N stage were the most important predictors for OS. The novel nomogram had superiority to the TNM staging system with improved C-index and IDI, as well as good consistency and higher clinical benefit. TDs are associated with poor survival from sigmoid colon cancer, and considering TDs can inform the formulation of individual treatment regimens. The nomogram shows satisfactory prediction ability for OS.

## Introduction

Colorectal cancer (CRC) is the third most common malignant tumor, and the second most common cause of cancer-associated mortality globally^1,2^. The sigmoid colon, which is located at the junction of the rectum and descending colon, is a site prone to colon cancer, with approximately 39.2% of colon cancers occurring here^3^. It has been reported that the prognosis of sigmoid colon cancer remains unsatisfactory, with a 5-year overall survival (OS) rate of 30–75%^4^. Because of individual differences, treatment responses differ among patients; these, in turn, create discrepancies in survival times. It is therefore important to provide individualized treatment strategies and to identify means of accurately predicting survival status.

Colorectal tumor deposits (TDs) are irregular cancer nodules that occur in the fat around the colon or rectum. These nodules are located in the lymphatic drainage of the primary cancer, but not in lymph node (LN) tissue, and lack vascular structure^5,6^. According to the 8th edition of the American Joint Committee on Cancer (AJCC) Cancer Staging Manual, patients who have sigmoid colon cancer with TDs and negative LNs are classified as N1c stage, while those with no TDs and negative LNs are classified as N0 stage. For patients with positive LNs, TD status is not considered in the N stage.

Existing studies have investigated the long-term survival of patients with CRC who have received chemotherapy, compared to those with and without TDs^7–9^; however, the conclusions of these studies have been inconsistent. This may be because the TD groups in the studies included patients with either positive or negative LNs; further, the researchers have thus far ignored tumor heterogeneity, including patients with rectal cancer and patients with colon cancer in their studies.

The present study examines data from the Surveillance, Epidemiology, and End Results (SEER) database and compares the survival rates of patients with stage N0 and N1c sigmoid colon cancer in terms of treatments received. It also explores the guiding role of TDs in treatment strategies. In addition, we apply a random forest (RF) machine learning algorithm and a nomogram to establish a prognostic model for predicting the survival of patients with sigmoid colon cancer. Through this approach, we found that TDs are an independent unfavorable prognostic factor for patients with sigmoid colon cancer, and that their presence/absence could guide decisions regarding treatment strategies. Further, our findings indicated that our model can help physicians more precisely estimate the survival of patients and, consequently, develop more appropriate individualized treatment plans.

## Materials and methods

### Patients

**Figure 1:**
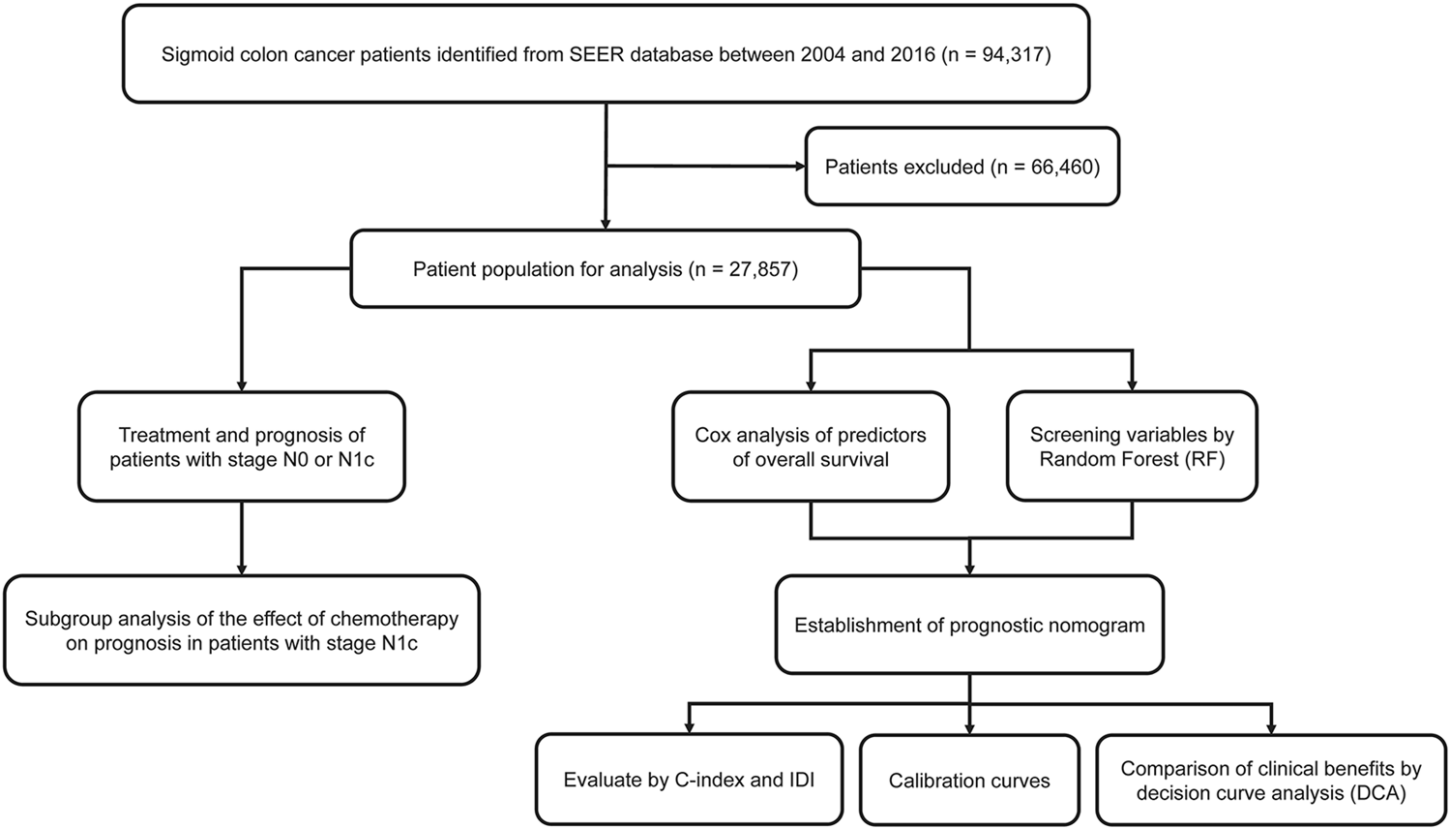
Flow chart of the overall study design.

We utilized the SEER database and SEER-stat software (SEER*Stat 8.3.9) to identify and collect data for patients who were diagnosed with pathologically confirmed sigmoid colon cancer between 2004 and 2016. Cases of sigmoid colon cancer were defined according to the International Classification of Diseases for Oncology (ICD-O-3). Through this search, 94,317 patients were preliminarily determined to meet the study conditions. Among these, 66,460 patients were excluded; the reasons for these exclusions were the following: (1) 24,969 patients had multiple primary tumors; (2) 15,694 patients were not treated with surgery or local tumor excision; (3) 46 patients’ diagnoses were not confirmed by pathology; (4) 17,454 patients had unknown T stage or N stage; (5) 7285 patients were in stage M1 or unknown; and (6) 1012 patients had a survival time of zero or had a negative follow-up. After these exclusions, the remaining 27,857 patients were included in the study group (Fig. 1).

### Definition of variables

For each patient, the T stage was restated to accord with the AJCC staging system (8th edition); this process was based on the following codes: derived AJCC T, 6th ed. (2004–2015); derived AJCC T, 7th ed. (2010–2015); and the Collaborative Stage (CS) extension (2004–2015)^10^. Meanwhile, the N stage was restated based on the following codes: derived AJCC N, 6th ed. (2004–2015); derived AJCC N, 7th ed. (2010–2015); regional nodes examined (1988+); regional nodes positive (1988+); and CS site-specific factor 4 (2004+ varying by schema). We then classified the data as follows: age at diagnosis was classified into three groups: < 50, 50–69, and ≥ 70 years old. Sex was classified as male or female, and race was classified into four categories: white, black, other, and unknown. Marital status was categorized as married, other, and unknown. Tumor differentiation was divided into three groups: well/moderately differentiated, poorly differentiated/undifferentiated, and unknown. Tumor size was classified into three categories: < 5 cm, ≥ 5 cm, and unknown^11,12^. Serum carcinoembryonic antigen (CEA) status was classified into three categories: elevated, normal, and unknown. Other clinical features were also included: perineural invasion (PNI) status (yes, no, and unknown), T stage (T1, T2, T3, T4a, and T4b), N stage (N0, N1a, N1b, N1c, N2a, and N2b), number of lymph nodes dissected (LND; ≥ 12, < 12, and unknown)^13,14^, received chemotherapy (yes and no), and received radiotherapy (yes and no).

### Statistical analysis and nomogram construction

The primary end result for this study was OS. Survival curves were generated using Kaplan–Meier curves and statistical comparisons were performed using the log rank test. The distribution of the T stage was measured using Likert scales. Multivariate Cox regression models were used to identify prognostic factors; a univariate Cox analysis was used to screen the factors (*p* < 0.05) in the multivariate Cox regression models.

RF was used to select factors, based on variable importance (VIMP). A positive VIMP value indicated that the variable can improve the predictive accuracy; the larger the value, the more obvious the effect^15^. RF is a regression tree technology with the advantages of a fast-training process, small estimation bias, and strong stability/anti-interference ability^16^. As it uses bootstrap resampling technology and selects feature sets through random sampling and random selection which can decrease the risk of the overfitting phenomenon and has a strong anti-interference capacity the model is forward-looking and can address nonlinear problems. Therefore, we adopted variable screening using RF to obtain more accurate predictions.

We developed a nomogram based on the RF results. Concordance index (C-index), integrated discrimination improvement (IDI), calibration curves, and decision-curve analysis (DCA) were used to evaluate the performance of the nomogram. The larger the C-index value, the more accurate the prediction^17^. IDI was used to compare the different models in terms of prediction accuracy^18^. Internal validation was performed using 1000 bootstrap resamples. The agreement between predicted survival and the actual survival was quantified using calibration curves of the nomogram for 1-, 3-, and 5-year OS. Evaluation of clinical usefulness and net benefit was performed using DCA^19^.

The data analysis was performed using SPSS version 26.0 for Windows (Armonk, NY: IBM Corp) and *R* software (version 3.6.3 and 4.0.3), utilizing the “survminer,” “survival,” “forestplot,” “randomForestSRC,” “rms” and “survIDINRI” packages. Statistical significance was set at *p* < 0.05.

### Ethical approval

The authors are accountable for all aspects of the work in ensuring that questions related to the accuracy or integrity of any part of the work are appropriately investigated and resolved. Institutional review board approval was waived for this study because SEER database is publicly available and de-identified. The author YB Lin has gotten the access to the SEER database (accession number: 12284-Nov2019). The authors are accountable for all aspects of the work.

## Results

### Patient characteristics

A total of 27,857 postoperative patients with sigmoid colon cancer were included in our analysis; their clinical and pathological characteristics are summarized in Table 1. Of these, 46.7% were female and 53.3% were male. The median age was 62 (interquartile range [IQR] 53–72) years. Patients with stage N1c accounted for 1.3% of the sample, and the median number of TDs was 1 (IQR 1–2). Chemotherapy was administered to 43.3% of the patients.

**Table 1:** Characteristics of the patients with sigmoid colon cancer.

Variable	Data, n (%)
Total	27,857 (100.0)
**Age (years)**	
< 50	4247 (15.2)
50–69	14,883 (53.4)
≥ 70	8727 (31.3)
**Gender**	
Male	14,855 (53.3)
Female	13,002 (46.7)
**Race**	
White	21,250 (76.3)
Black	2892 (10.4)
Other	3535 (12.7)
Unknown	180 (0.6)
**Marital status**	
Married	15,832 (56.8)
Other	10,741 (38.6)
Unknown	1284 (4.6)
**Tumor differentiation**	
Well/moderately differentiated	23,256 (83.5)
Poorly differentiated/undifferentiated	3797 (13.6)
Unknown	804 (2.9)
**Tumor size (cm)**	
< 5	16,863 (60.5)
≥ 5	8778 (31.5)
Unknown	2216 (8.0)
**Serum CEA**	
Elevated	5809 (20.9)
Normal	10,451 (37.5)
Unknown	11,597 (41.6)
**Perineural invasion**	
Yes	1914 (6.9)
No	17,203 (61.8)
Unknown	8740 (31.4)
**T stage**	
T1	4545 (16.3)
T2	3971 (14.3)
T3	15,409 (55.3)
T4a	2492 (8.9)
T4b	1440 (5.2)
**N stage**	
N0	12,066 (43.3)
N1a	5099 (18.3)
N1b	5015 (18.0)
N1c	462 (1.7)
N2a	3038 (10.9)
N2b	2177 (7.8)
**Number of lymph nodes dissected**	
≥ 12	21,807 (78.3)
< 12	6050 (21.7)
**Chemotherapy**	
Yes	12,066 (43.3)
No	15,791 (56.7)
**Radiotherapy**	
Yes	830 (3.0)
No	27,027 (97.0)

*CEA* carcino-embryonic antigen.

### Survival of patients with stage N0 or stage N1c sigmoid colon cancer

**Figure 2:**
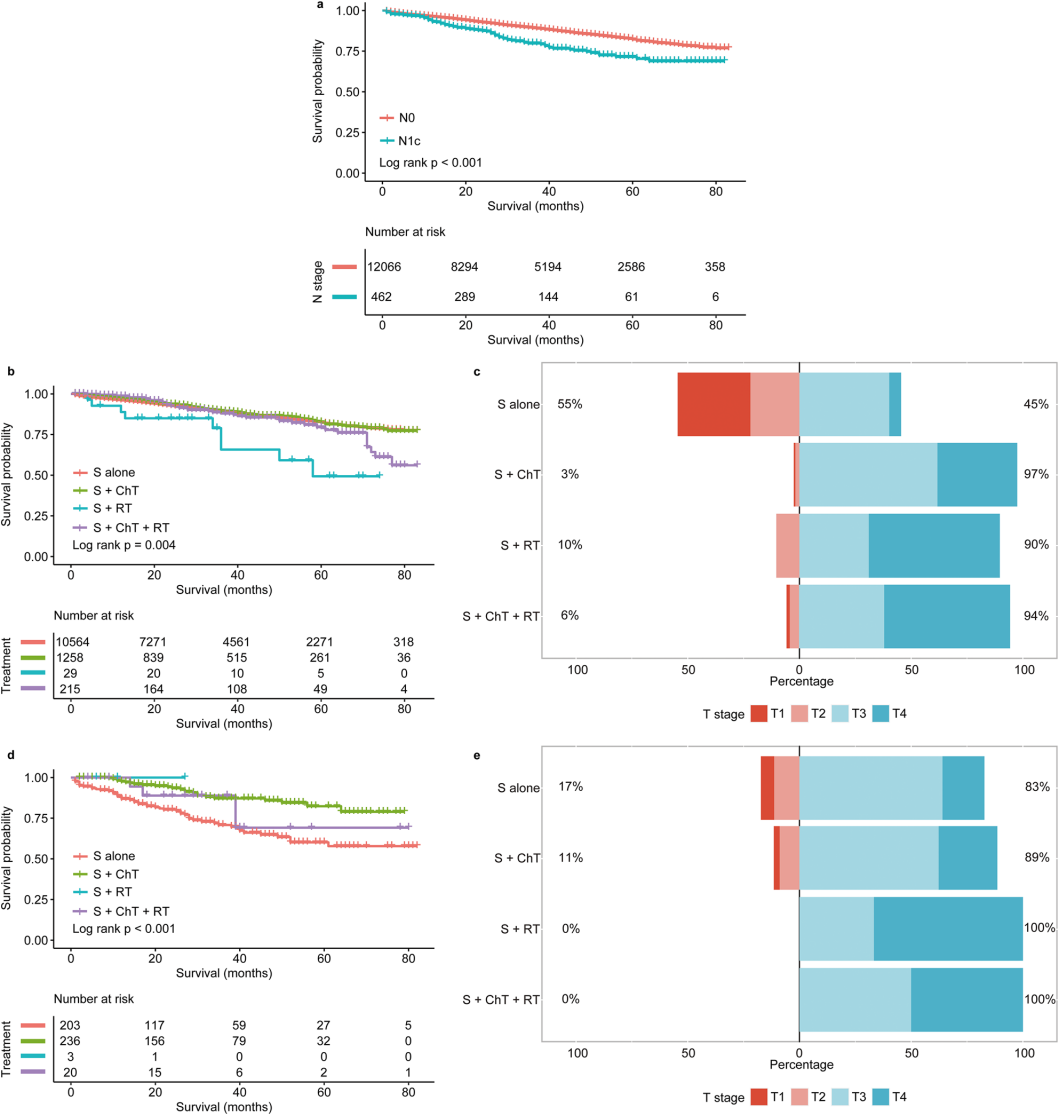
Overall survival of patients with stage N0 and stage N1c sigmoid colon cancer. (**a**) Kaplan–Meier estimates for comparison of OS between the N0 and N1c groups. (**b**, **d**) Effect of treatment modalities on the OS of the N0 and N1c groups. (**c**,** e**) Distribution of T stage across the different treatment groups in the N0 and N1c groups. The number of at-risk cases in each group at months 0, 20, 40, 60, and 80 are indicated. *S alone* Surgery alone, *S* surgery, *ChT* chemotherapy, *RT* radiation.

As shown in Fig. 2a, stage N0 patients had a better prognosis than stage N1c patients (*p* < 0.001); the 1-, 3-, and 5-year OS rates for these two groups were 96.14% versus 93.08%, 89.19% versus 79.76%, and 82.20% versus 71.46%, respectively. The restricted mean survival time of the two groups was 72.0 (95% confidence interval [CI] 62.6–68.7) months and 65.7 (95% CI 71.6–72.5) months, respectively (*p* < 0.001). And the competing risk model showed no statistical difference in non-tumor mortality between the stage N0 and N1c groups (*p* = 0.957), indicating that the difference in OS between the two groups was mainly due to the tumor (*p* < 0.001) (Supplementary Fig. 1).

The survival of stage N0 and N1c patients after receiving different treatments is shown in Fig. 2b,d. For stage N0 patients, surgery combined with chemotherapy or radiotherapy did not improve survival, compared with surgery alone (*p* = 0.456, *p* < 0.001, respectively); this was despite the fact that the proportions of patients with stage T3 and T4 tumors were higher in the two former groups (i.e., chemotherapy and radiotherapy, respectively; Fig. 2c). Meanwhile, for stage N1c patients, the survival rate of the surgery plus chemotherapy group was significantly higher than that of the only surgery group (*p* < 0.001); the distribution of T stage was similar among these two groups (Fig. 2e).

**Figure 3:**
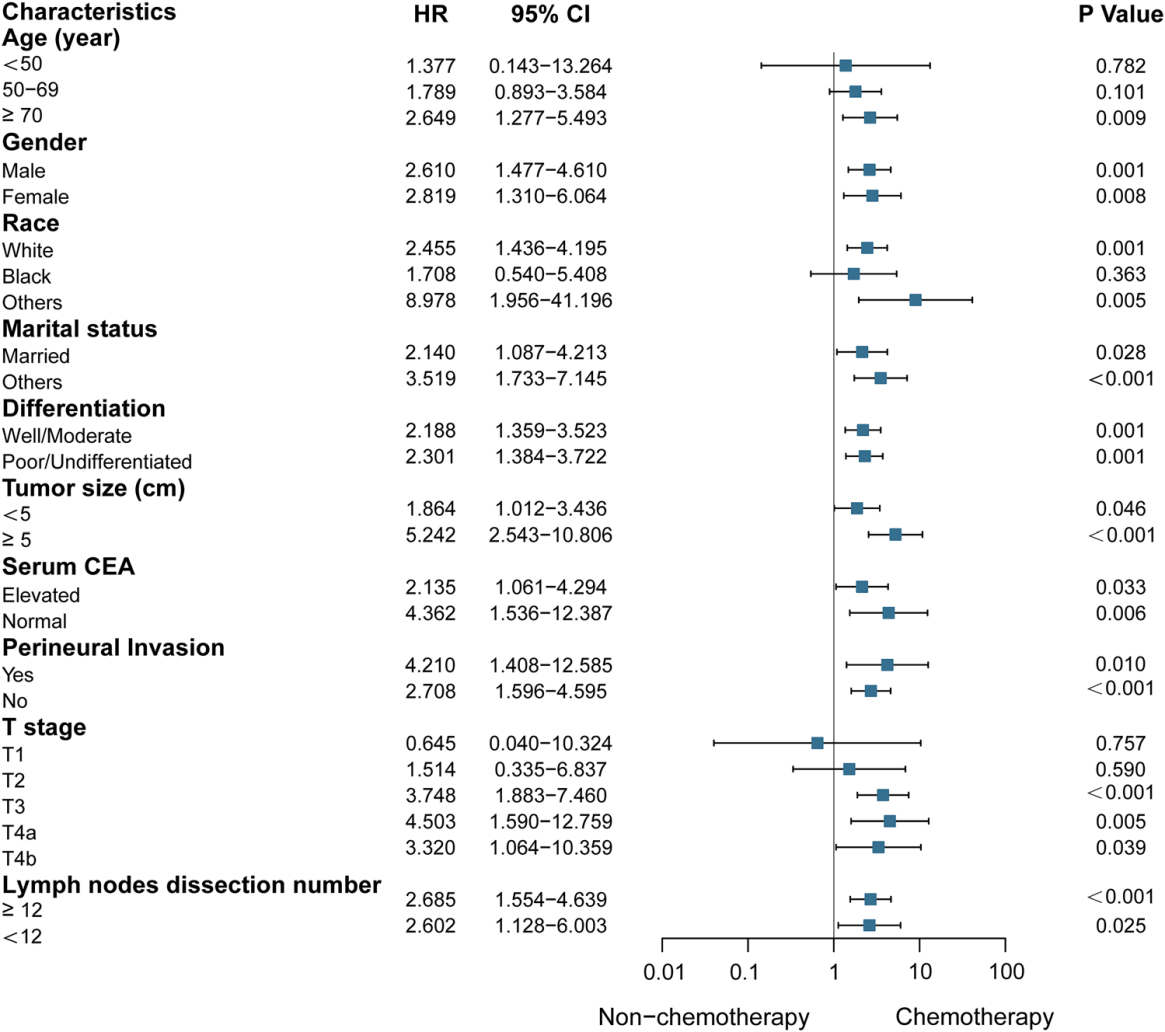
Subgroup analysis of the different factors for the N1c group (chemotherapy versus non-chemotherapy, hazard ratio ± 95% confidence interval). *CEA* Carcino-embryonic antigen, *CI* confidence interval, *HR* Hazard ratio.

The subgroup analysis showed the survival rates of stage N1c patients who did and who did not receive chemotherapy, respectively (Fig. 3). Here, chemotherapy showed no effect on the prognosis of patients who were younger than 70 years, those with stage T1–2, and those of black race (all *p* > 0.05). Other patients benefited from chemotherapy (all *p* < 0.05).

### Prognostic factors affecting OS

**Figure 4:**
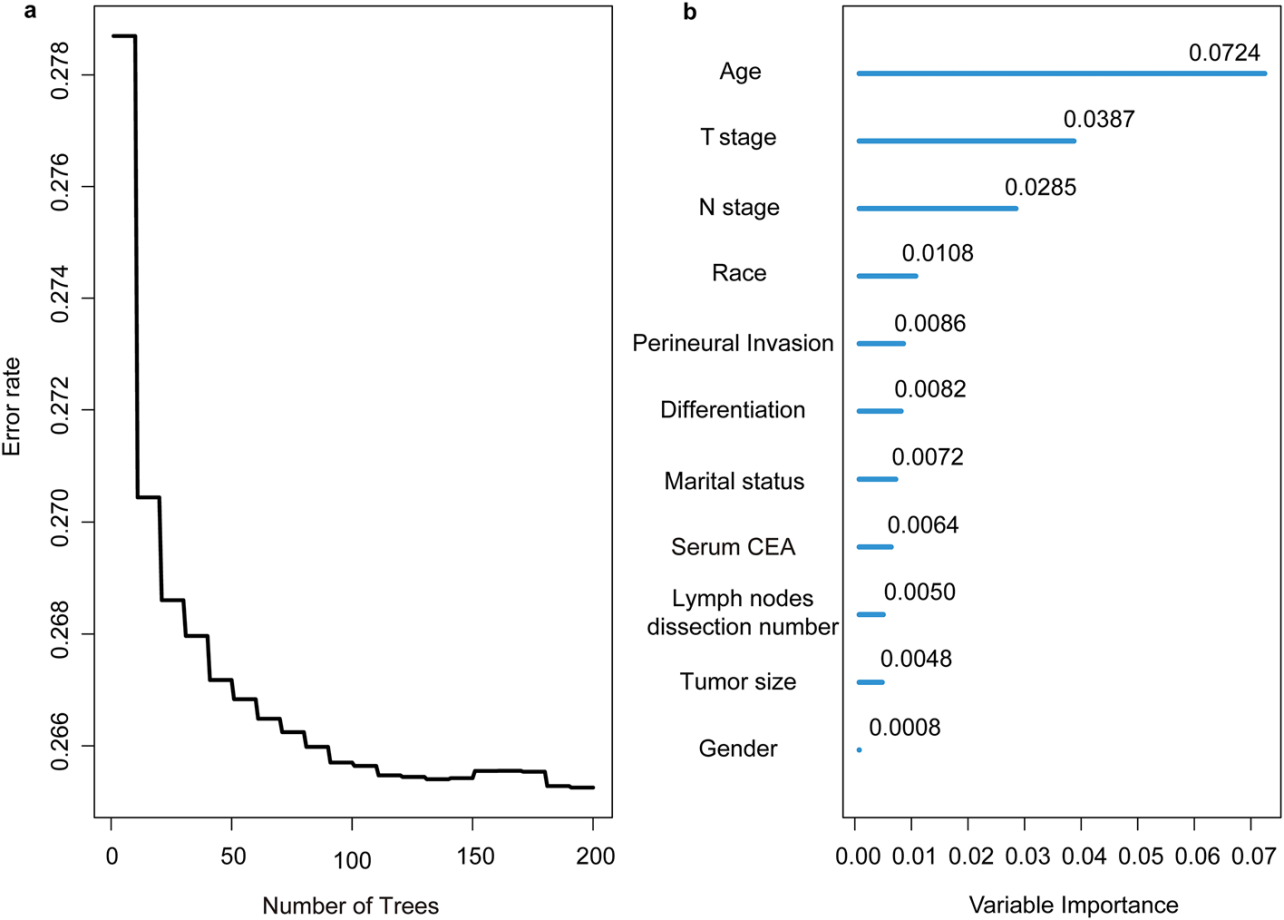
Prediction error for RF and VIMP values for each variable among sigmoid colon cancer patients. (**a**) Prediction error rates for cumulative hazard function. (**b**) The VIMP values for each variable. *CEA* Carcino-embryonic antigen, *VIMP* variable importance.

The univariate and multivariate Cox regression analyses revealed that the independent predictors of OS were age, sex, race, marital status, tumor differentiation, tumor size, serum CEA, PNI, T stage, N stage, and LND number (Table 2). Compared with stage N0 patients, stage N1c patients had a 1.310 times higher risk of death (95% CI 1.049–1.638, *p* = 0.017). The results of the RF are presented in Fig. 4, including the relationship between the number of trees and the prediction error rate, and the VIMP of each factor in terms of OS. The prediction error tends to be stable if the number of trees in the forest exceeds 100 (Fig. 4a). Age, T stage, and N stage were the most important predictors associated with OS (VIMP = 0.0724, 0.0387, and 0.0285, respectively); other factors had relatively small and similar effects on OS (Fig. 4b).

**Table 2:** Variables associated with overall survival, according to the Cox proportional hazards regression model.

Variable	Univariable analysis		Multivariable analysis	
	Hazard ratio (95% CI)	*p* value	Hazard ratio (95% CI)	*p* value
**Age (year)**		< 0.001		< 0.001
< 50	Reference	–	Reference	–
50–69	1.158 (1.062–1.262)	0.001	1.292 (1.185–1.409)	< 0.001
≥ 70	3.428 (3.152–3.727)	< 0.001	3.680 (3.381–4.005)	< 0.001
**Gender**				
Male	Reference	–	Reference	–
Female	0.880 (0.838–0.923)	< 0.001	0.777 (0.739–0.817)	< 0.001
**Race**		< 0.001		< 0.001
White	Reference	–	Reference	–
Black	1.222 (1.135–1.316)	< 0.001	1.234 (1.145–1.330)	< 0.001
Other	0.857 (0.793–0.927)	< 0.001	0.842 (0.779–0.911)	< 0.001
Unknown	0.240 (0.120–0.481)	< 0.001	0.287 (0.143–0.574)	< 0.001
**Marital status**		< 0.001		< 0.001
Married	Reference	–	Reference	–
Other	1.623 (1.546–1.705)	< 0.001	1.453 (1.381–1.529)	< 0.001
Unknown	1.055 (0.926–1.202)	0.419	1.083 (0.950–1.234)	0.233
**Tumor differentiation**		< 0.001		< 0.001
Well/moderately differentiated	Reference	–	Reference	–
Poorly differentiated/un-differentiated	1.552 (1.461–1.647)	< 0.001	1.237 (1.163–1.316)	< 0.001
Unknown	0.732 (0.618–0.867)	< 0.001	1.031 (0.868–1.226)	0.727
**Tumor size (cm)**		< 0.001		< 0.001
< 5	Reference	–	Reference	–
≥ 5	1.423 (1.353–1.496)	< 0.001	1.162 (1.102–1.226)	< 0.001
Unknown	0.653 (0.586–0.729)	< 0.001	0.994 (0.885–1.117)	0.925
**Serum CEA**		< 0.001		< 0.001
Elevated	Reference	–	Reference	–
Normal	0.495 (0.464–0.528)	< 0.001	0.672 (0.630–0.718)	< 0.001
Unknown	0.736 (0.695–0.780)	< 0.001	0.909 (0.857–0.964)	0.002
**Perineural invasion**		< 0.001		< 0.001
Yes	Reference	–	Reference	–
No	0.508 (0.461–0.561)	< 0.001	0.811 (0.732–0.898)	< 0.001
Unknown	0.832 (0.755–0.918)	< 0.001	0.876 (0.793–0.969)	0.010
**T stage**		< 0.001		< 0.001
T1	Reference	–	Reference	–
T2	1.571 (1.380–1.789)	< 0.001	1.374 (1.200–1.574)	< 0.001
T3	2.98 (2.690–3.313)	< 0.001	2.03 (1.808–2.280)	< 0.001
T4a	5.366 (4.769–6.038)	< 0.001	3.408 (2.990–3.883)	< 0.001
T4b	6.018 (5.293–6.843)	< 0.001	3.766 (3.270–4.337)	< 0.001
**N stage**		< 0.001		< 0.001
N0	Reference	–	Reference	–
N1a	1.601 (1.487–1.725)	< 0.001	1.345 (1.237–1.463)	< 0.001
N1b	1.888 (1.757–2.030)	< 0.001	1.548 (1.423–1.683)	< 0.001
N1c	2.236 (2.066–2.420)	< 0.001	1.310 (1.049–1.638)	0.017
N2a	3.240 (2.989–3.513)	< 0.001	1.778 (1.622–1.949)	< 0.001
N2b	1.776 (1.424–2.216)	< 0.001	2.606 (2.370–2.865)	< 0.001
**Number of lymph nodes dissected**				
≥ 12	Reference	–	Reference	–
< 12	1.312 (1.245–1.382)	< 0.001	1.469 (1.390–1.552)	< 0.001

*CI* confidence interval, *CEA* carcino-embryonic antigen.

### Establishing the prognostic nomogram model

**Figure 5:**
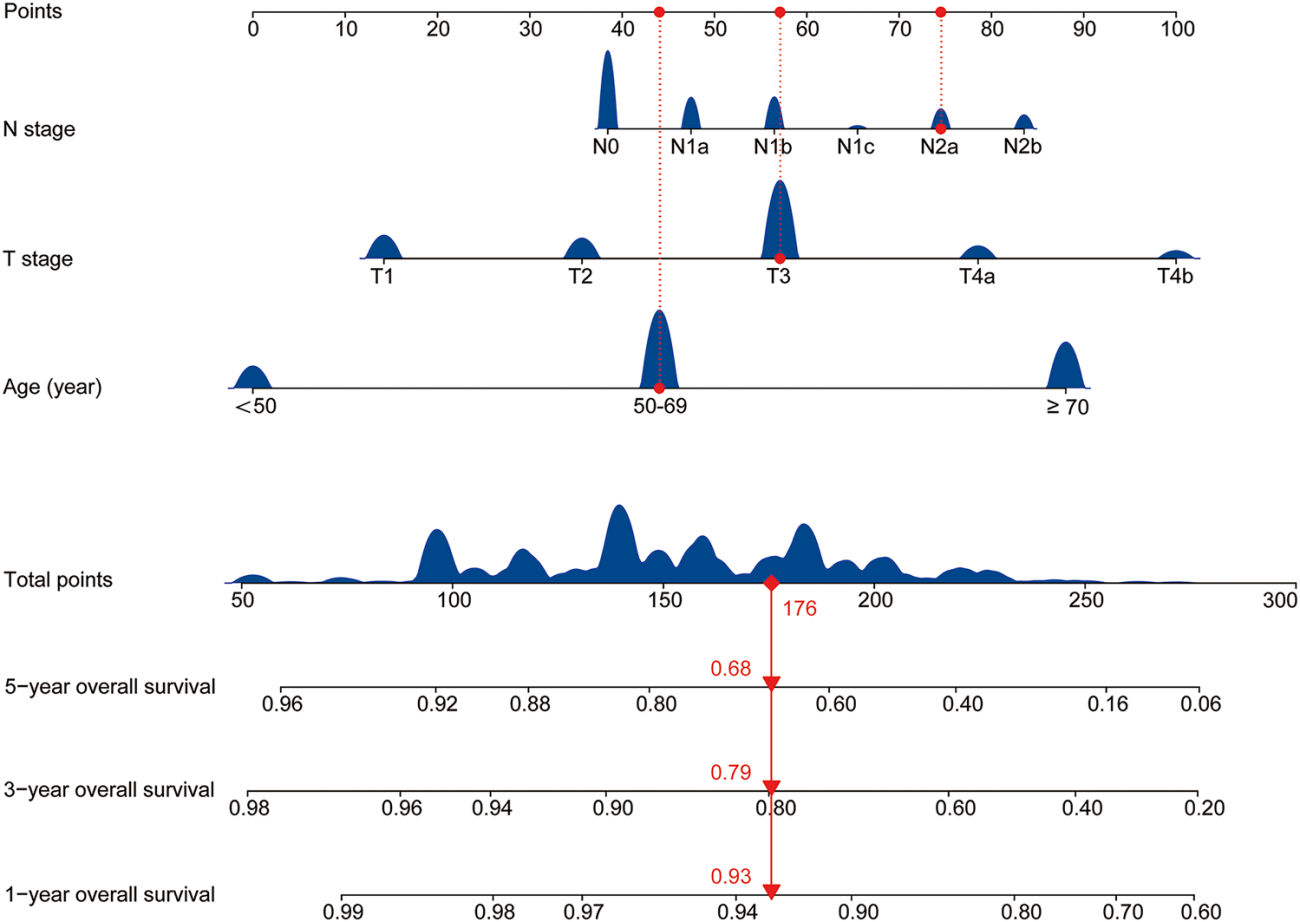
The 1-, 3- and 5-year OS probability is calculated by determining the sum of the risk points, which are based on age, T stage, and N stage. For each parameter, the number of associated risk points can be determined by drawing a vertical line from each variable axis upwards to the points axis (0–100). The total score projected on the bottom scale represents the probability of OS rates of 1-, 3- and 5-years. *OS* Overall survival.

Based on the results of the RF, a nomogram was constructed to use age, T stage, and N stage to predict the 1-, 3-, and 5-year OS of patients with sigmoid colon cancer (Fig. 5). Most patients scored between 50 and 250 points. Compared with the AJCC’s TNM staging system, the nomogram showed a high C-index (C-index for nomogram = 0.728, 95% CI 0.721–0.734; C-index for AJCC TNM staging system = 0.667, 95% CI 0.660–0.675, *p* < 0.001); the IDI was 0.074 (95% CI 0.066–0.081, *p* < 0.001).

**Figure 6:**
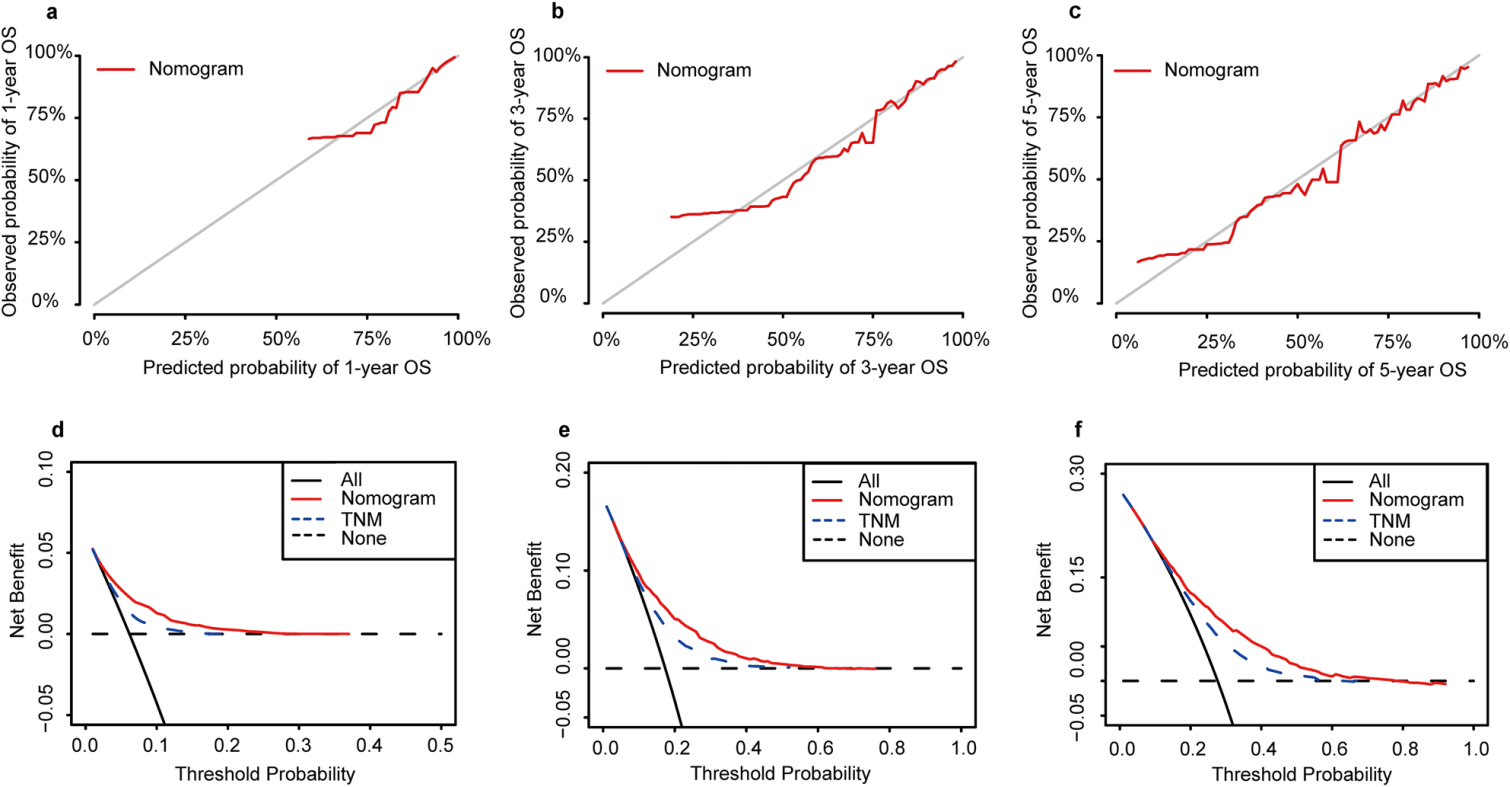
Development and performance of the nomogram. Calibration curves to assess the 1- (**a**), 3- (**b**), and 5- (**c**) year OS rates. Decision curves compare nomogram and TNM stages for 1- (**d**), 3- (**e**), and 5- (**f**) year OS rates. The red line represents the nomogram; the black line represents the TNM stage. *OS* Overall survival.

The calibration plots demonstrated good concordance between the actual 1-, 3-, and 5-year OS rates and the survival rates predicted by the nomogram (Fig. 6a–c). As shown in Fig. 6d–f and based on the threshold probability (x-axis) and net benefit (y-axis), the DCA indicated that the model was clinically useful. The results indicated that the new nomogram had better net benefits than the AJCC TNM model for the first, third, and fifth years.

## Discussion

This study revealed that the presence of TDs is an independent, unfavorable prognostic factor for patients with sigmoid colon cancer (hazard ratio [HR] 1.310, 95% CI 1.049–1.638, *p* = 0.017), and that their presence/absence could guide decisions regarding treatment strategies. Not all stage N1c patients should receive chemotherapy, which is recommended for positive LN patients. Patients aged < 70 years, those of black race, and those with stage T1–2 showed similar curative prognoses when receiving surgical treatments alone, as when receiving a combination of surgery and chemotherapy (all *p* > 0.05). Thus, patients without other high-risk factors may be able to forgo chemotherapy in clinical practice. Using the results of multivariable analysis and VIMP values obtained through RF, factors that have a significant impact on OS were selected to establish a nomogram model for predicting one-, three-, and five-year OS rates. The new model had better discrimination than the AJCC’s TNM model (C-index: 0.728 versus 0.667, respectively, *p* < 0.001; IDI = 0.074, *p* < 0.001), as well as good consistency and higher clinical benefit.

The National Comprehensive Cancer Network’s guidelines suggest that TDs are related to the N stage. However, some studies have suggested that patients with stage N1c can be incorporated into stage N1b or stage N2a groups, in terms of their survival^20,21^. Some studies also show that the N stage can be further subdivided by counting TDs as positive LNs^7,22^. Our study demonstrated that patients with stage N1c have worse OS than patients with stage N0 (*p* < 0.001). Further, our multivariable analysis revealed that patients with stage N1c or stage N1a have a similar risk of death as those with stage N0 (HR 1.310, 95% CI 1.049–1.638; HR 1.345, 95% CI 1.237–1.463). These results indicate that the role of TDs in sigmoid colon cancer is complicated and that they may play important roles in tumor proliferation and invasion.

Nevertheless, the exact effect and mechanism of TDs in prognosis remain unclear. Ueno find that the most prominent feature of TDs is undifferentiated cancer cell microscopic clusters in adipose tissue, which is similar to tumor budding at the invasive front of the main tumor^23^. Numerous studies have shown that tumor budding may be the early stage of invasion and related to LN infiltration and poor prognosis^24,25^. Therefore, TDs may also indicate that a tumor is more invasive. In addition, Prabhudesai find a marked association between TDs and vascular invasion, suggesting that TDs represent bloodborne spread and an early indicator of distant metastasis of tumor cells^26^. Therefore, it is reasonable to conclude that patients with TDs will have a worse prognosis.

We obtained new insights regarding therapeutic strategies for patients with stage N1c disease without distant metastasis. Surgery combined with adjuvant chemotherapy is recommended for patients with stage III colon cancer. Bouquot find that stage N1c is associated with a higher T stage, and suggests that such patients be considered high-risk and candidates for adjuvant chemotherapy^27^. However, the present study found that not all patients with stage N1c sigmoid colon cancer require chemotherapy following surgery. For patients younger than 70 years, those with stage T1–2 and those of black race receiving chemotherapy did not impact prognosis (all *p* > 0.05). If patients with these characteristics do not have high-risk factors—such as positive resection margins, neurovascular invasion, or vascular tumor thrombus—surgery alone, and enhanced follow-up monitoring, could be considered to thereby avoid subjecting patients to unnecessary treatment. Previous studies had shown that older adults have poor resistance to chemotherapy and cannot benefit from it. Nevertheless, it has been shown that older adult patients (≥ 75 years old) with stage III colon cancer can benefit from adjuvant chemotherapy compared to those who did not receive adjuvant chemotherapy (3-year overall survival: 60–71% versus 50–53%)^28^. The International Society of Geriatric Oncology (SIOG) also believed that older adult patients with stage III colon cancer can obtain the same benefits as young people from adjuvant chemotherapy^29,30^. Therefore, for N1c stage III older adult patients, after evaluating chemotherapy tolerance, adjuvant chemotherapy may be considered to obtain better survival rates.

For patients with stage N0, receiving surgery combined with chemotherapy showed no impact on OS, compared to receiving surgery alone (*p* = 0.456). This is similar to the findings of other studies. The British Quick and Simple and Reliable trial (commonly referred to as the QUASAR trial) and a previous meta-analysis conclude that the 5-year OS benefit of fluorouracil-based adjuvant chemotherapy is only 3–4% (95% CI 1.0–6.0), suggesting that it fails to significantly improve the prognosis of patients with stage II colon cancer^31,32^. Further, the MOSAIC trial indicated that adjuvant oxaliplatin-based chemotherapy does not provide survival benefits for patients with stage II colon cancer, unless they have high-risk clinical features^33^.

For both stage N0 and N1c patients, our study did not find a prognostic benefit of radiotherapy. Currently, the available data on whether radiotherapy can increase OS rates in patients with colon cancer is inconsistent^34^. Retrospective studies from the 1980s and 1990s have demonstrated improvements in local control (LC) and disease-free survival (DFS) after adjuvant external beam radiotherapy^35,36^. However, in a subsequent randomized controlled trial, no differences in DFS or OS were observed between the test groups, which was inconsistent with the previous conclusions^37^. Meanwhile, a recent retrospective study reports that adjuvant radiotherapy can improve LC and DFS in some patients with colon cancer, such as those with stage T4b and/or residual tumors^38^. However, organ movement is an important factor affecting the accuracy of radiotherapy. Some studies indicate that respiratory movement seriously affects the effect of radiotherapy on CRC tumors, which increases the difficulty of using radiotherapy to treat colon cancer^39,40^. Currently, the clinical application of radiotherapy in colon cancer is insufficient, meaning there is a lack of data concerning exposure ranges, radiation dosages, and fraction numbers; this lack of data contributes to the limited application of radiotherapy, forming a vicious circle. Therefore, the role of radiotherapy in colon cancer requires further investigation.

As sigmoid colon cancer has a poor prognosis, it is important to determine prognostic factors. Previous researches find that age, tumor differentiation, T stage, and N stage are independent factors influencing survival, of which T stage is the most significant factor^41,42^. The present study also confirmed the role of these factors in prognosis, using multivariable analysis. Meanwhile, we also found sex, race, marital status, serum CEA level, tumor size, PNI, and LND number to be independent predictors of OS (all *p* < 0.05), suggesting that there are numerous factors affecting the survival of patients with sigmoid colon cancer. Therefore, it is necessary to determine key prognostic characteristics. We calculated and ranked the influence of each factor on survival through the machine learning method RF and found that the effects of age, T stage, and N stage on survival are greater than those of other factors (VIMP = 0.0724, 0.0387, and 0.0285, respectively). Benefiting from the high stability of RF-calculated results, the established prediction model had good generalization ability.

Furthermore, we constructed a nomogram based on age, T stage, and N stage to predict the 1-, 3-, and 5-year OS of patients with sigmoid colon cancer. To our knowledge, this is the first predictive model for sigmoid colon cancer survival. The nomogram showed that the scores for the different age, T stage, and N stage groups had large ranges, suggesting that these factors have a greater impact on prognosis. Among these, age showed the greatest influence on OS; this may be related to the limited remaining life of older adults, as well as their poor tolerance of extensive surgery, radiotherapy, and chemotherapy. As an example for explaining the application of the model, consider a patient with sigmoid colon cancer who is aged 61 years (43 points), and is postoperative stage T3 (58 points), N2a (75 points), and M0. For this patient, the total risk value would be 176, trending downward on the “1-, 3-, and 5-year overall survival” axes. The 1-year survival rate would be 93.0%, the 3-year survival rate would be 79.0%, and the 5-year survival rate would be 68.0%.

In addition, the nomogram demonstrated better discrimination in predicting OS, compared to the TNM staging system, showing a C-index of 0.728 in comparison to the TNM’s C-index of 0.667 (*p* < 0.001). IDI was 0.074 (*p* < 0.001), indicating that the predictive capability of the new model was improved by 7.40%. For internal validation, bootstrap resampling with 1000 iterations was performed, and the calibration plots exhibited good consistency between the predicted and actual OS. Finally, the DCA for the first, third, and fifth years showed that, in clinical practice, predicting the survival of patients using this novel nomogram can bring higher benefits for patients than using the TNM staging model. This may be because different expected survival times can cause different psychological burdens for patients, and can also have an impact on subsequent treatment and follow-up strategies.

There are some limitations to this study. First, the existence of missing data in samples inevitably introduced a potential selection bias and accuracy loss. Second, because of the limitations of the SEER database, some information was not included, including tumor recurrence, details on chemotherapy regimens, and radiation plans. Third, the SEER database lacks information such as surgical margin status, microsatellite status, and Ki-67 proliferation index; therefore, these variables are not included in the prediction model. Fourth, it is important to highlight that the nomogram created in this study only applies to patients without distant metastasis and who had undergone surgery. Lastly, this was a retrospective study, and further prospective, multicenter, large-scale studies are needed to verify our findings.

## Conclusion

The present study’s findings suggest that TDs indicate poor survival among patients with sigmoid colon cancer, and their presence/absence should be considered when formulating individual treatment regimens. Patients with stage N1c have a worse prognosis than those with stage N0 disease. It may be possible to alter treatment plans to avoid the administering of adjuvant chemotherapy to stage N1c patients who are younger than 70 years, of black race, and/or with stage T1–2. The nomogram model created in this study showed good prediction accuracy and consistency. This model could help physicians more precisely estimate the survival of patients, and consequently develop individualized treatment plans that provide better clinical benefits for patients.

## Supplementary Information

Supplementary Figure S1.

## Data Availability

The datasets used and/or analyzed during the current study are available from the corresponding author on reasonable request.
