# Supplementary material for: Identifying the long-term survival beneficiary of chemotherapy for stage N1c sigmoid colon cancer
Source: Sci Rep. 2022 Oct 7;12:16909. doi: 10.1038/s41598-022-21331-z (PMC9546836; doi:10.1038/s41598-022-21331-z)
Supplement: Supplementary file 1 — Supplementary Figure S1. [file 41598_2022_21331_MOESM1_ESM.docx]

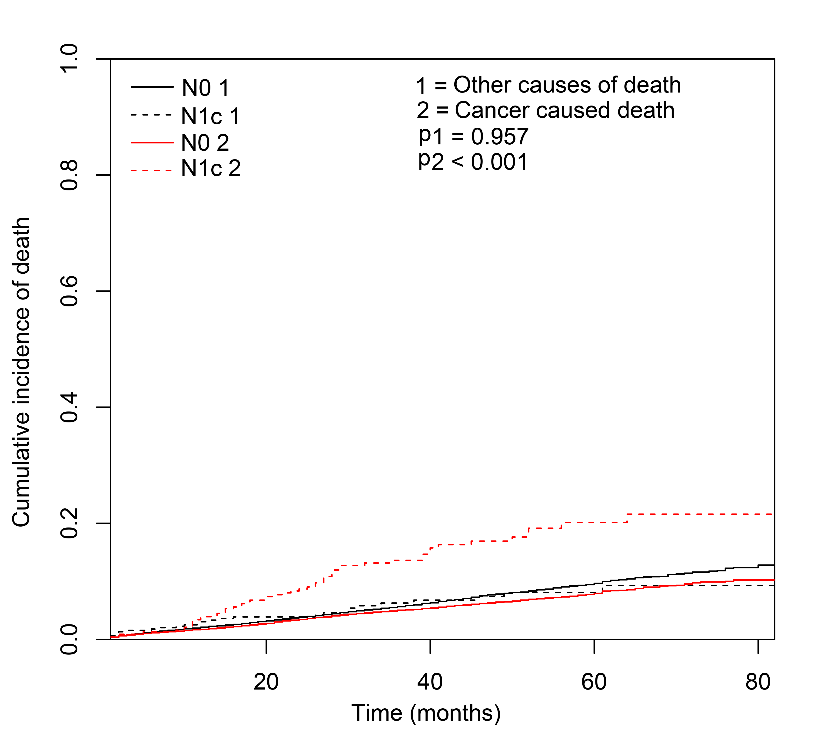


**Supplementary Fig. 1** Cumulative incidence plot depicting cancer-caused death and other cause mortality according to the N0 and N1c groups.
